# Integration of artificial intelligence-based solutions into electronic gaming machines for responsible gambling: a case study of South Africa

**DOI:** 10.3389/frai.2026.1715864

**Published:** 2026-07-09

**Authors:** Daniel Makhubela, Ilesanmi Daniyan, Jan Adrian Swanepoel, Lanre Daniyan, Adefemi Adeodu, Humbulani Simon Phuluwa

**Affiliations:** 1 Department of Industrial Engineering, Tshwane University of Technology, Pretoria, South Africa; 2 Department of Mechatronics Engineering, Bells University of Technology, Ota, Nigeria; 3 Department of Industrial Engineering & Engineering Management, University of South Africa, Florida, South Africa; 4 Centre for Space Earth Station and Observatory (CSESO) Eruwa, Oyo State, Nigeria; 5 Department of Project Management, Bells University of Technology, Ota, Nigeria

**Keywords:** artificial intelligence (AI), EGM, gambling, harm minimization, pattern recognition and classification model

## Abstract

The increase in sophisticated electronic gambling products offered by gambling manufacturers facilitates an increase in the accessibility of gambling platforms and modes. This study proposes utilising Artificial Intelligence (AI) to enhance self-awareness and self-control within electronic gaming machines. The study adopts a case study that follows an exploratory research design, as the causal explanation argument suggests that the Electronic Gaming Machine (EGM) device causes problem gambling. Secondary data on gambling were obtained and analysed. The machine-learning pattern recognition and classification model; a learning approach which learns from a trained dataset to make decisions or predictions, was employed. The dataset was trained iteratively using the scaled conjugate gradient backpropagation with the input and output target samples divided into training, validation, and test datasets. Furthermore, the softmax was used for classifying the dataset into three classes: responsible, intermediate, and irresponsible gambling. The confusion matrix was used to analyse the percentages of correct and incorrect classifications. The results obtained indicated that the accuracy of the developed model was 99.20%, while the precision was 85.70%. The recall achieved 85.70%, while the F1-score reached 80.50%. The closeness of these performance indices to 1, coupled with the negligible value of mean square error, indicates that the developed classification model is robust and suitable for classification problems. Thus, this study contributes to knowledge by developing an AI model that can track players and reduce harm in a land-based gambling environment.

## 1 Introduction

The accessibility of this product encourages rapid gameplay or continuous gameplay (gambling). This continuous gameplay on the device poses a severe challenge to the responsible gambling Programme. Gambling devices can keep players engaged on the device or facilitate a loss of control while they are using it. This research develops an appropriate artificial intelligence (AI)-based model that can control gambling problems, benefiting both gaming operators and responsible gambling organisations. According to Nzimande et al. (2010), gambling in South Africa was legalised in 1996 with the enactment of the National Gambling Act. This act established regulations for gambling activities and created uniform standards for all gambling entities. Among the various forms of gambling, Electronic Gaming Machine (EGM) gambling in casinos emerged as the most popular and safest option, thanks to the stringent regulations imposed by the National Gambling Act.

The National Gaming Board (NGB) oversees the regulation of the national gambling industry. It upholds South Africa’s integrity as a responsible global citizen while examining the social and economic impacts of gambling on society (National Gambling Board, 2022). Walker and Sobel (2016) emphasised that the social and economic impact of gambling dates back to the early 1990s. In the United States of America, the development of this literature primarily focuses on the growth of the Casino industry. In contrast, in South Africa (S. A.), it encompasses the entire gambling industry. Research conducted by Anderton (2016) indicates that the Gross Gaming Revenue (GGR) of slot machines in the United States has declined over the past few years, as most revenue is generated from slot machine play.

According to National Gambling Board (2022), the Gross Gaming Revenue (GGR) of Casinos in South Africa has dropped from 56% in the financial year 2019–2020 (FY2019–2020) to 39.20% in FY2020–2021, whereas betting has increased by 26 to 45% from FY2019–2020 to FY2020–2021.

The National Gambling Board (2022) report shows a trend of Electronic Gaming Machine or table gameplay revenue drops, which can be traced back to 2010, when it was at 84.4 to 39.20%. For 10 years, it lost 45.2% of its revenue, while betting increased from 10 to 45.6%.

The trend of Gross Gaming Revenue (GGR) in the casino market has been steadily declining over the past decade, whereas sports betting has experienced a notable increase. As of March 31, 2016, the number of licensed operators across various gambling modes (excluding the National Lottery) in South Africa. The range of licensed holders includes 38 operational casinos and 2,072 LPM site operators. While the number of licensed casino operators has remained constant, the number of electronic gaming machines increased by 18,953 during the 2020–2021 financial year (National Gambling Board, 2022). Casino operators in South Africa have state-of-the-art buildings that attract people for entertainment. At the same time, electronic gaming devices, also known as electronic gaming machines, are the primary drivers of that entertainment. The National Responsible Gambling Program (NRGP) is an entity that plays a vital role in gambling, and it provides treatment for gambling-related problems.

Harris (2019) describes the intensity and accessibility of electronic gambling machines, and a significant number of patients originate from casino environments. These findings suggest that electronic gaming machines contribute to a greater percentage of problem gambling within the gambling industry. The report further details that the instrument employed to assess gambling-related harm is the Canadian Problem Gambling Index. Dowling et al. (2005) assert that EGMs are disproportionately represented as the predominant form of gambling reported by problem gamblers who seek treatment. This is mainly due to the inherent structural characteristics of EGM.

The South African Responsible Gambling Foundation’s annual report for 2017/2018 presents a demographic analysis of irresponsible gamblers in South Africa, indicating a higher prevalence among males than females who participate in the treatment programme. The report highlights that the issue is primarily associated with electronic gambling machines. Njobe (2013) argues that various strategies are available to reduce problem gambling worldwide. The author proposes a harm minimisation approach, which includes limiting the time spent on gaming devices without breaks and adjusting deposit limits according to the denomination of the machine.

This research aims to develop an artificial intelligence-based model for classifying gambling activities into responsible, intermediate and problem or irresponsible gambling in electronic gambling systems. It encompasses a review literature on EGM to understand the perspective of responsible gambling limit-setting tools regarding specific gambling problems and the development of AI-based model and framework for promoting responsible gambling as well as the provisions of recommendations based on the outcome of the study.

This study aligns with the broader of Sustainable Development Goals (SDG) goal 3 which emphasizes good health and social well-being. The SDG 3 aims to strengthen the prevention and treatment of substance abuse, including harmful use of alcohol and drugs (United Nations Department of Economic and Social Affairs, 2025), which could be extended to include gambling addiction.

This study is significant in that it contributes to the promotion of safety. This study addresses the integrity of gambling operators and proposes various harm minimisation strategies to enhance the entertainment value of gambling. By implementing the AI model developed in this research, we aim to alleviate the adverse effects of irresponsible gambling, such as unhealthy emotions, excessive time spent gambling, surpassing betting limits, and issues like depression, stress, and anxiety. When gamblers adopt a more responsible mindset and approach to betting, it can lead to greater happiness and health within families, as well as a more productive and safer society, reducing social issues such as crime and suicide attempts.

Furthermore, this research seeks to raise awareness and enhance understanding of problem gambling. The artificial intelligence system currently in use is effectively designed to deliver the necessary outcomes for addressing this issue. This study also recommends incorporating harm minimisation strategies, such as encouraging players to take breaks, alongside integrating features like automated anxiety and agitation monitoring devices to foster responsible gambling practices.

## 2 Literature review

### 2.1 Gambling

Whelan et al. (2007) defined gambling as an activity that involves risk-taking and betting on the outcome using a commodity of value or betting on the event with the chance of getting the desired result. Whether it is in casinos, Bingo, limited-pay-out machines (LPM), lotteries, or Bookmakers, nine provinces now have legalised gambling activities through the Provincial Licensing Authority (PLA).

Friedl (2022) asserts that the emergence of the internet and progressively advanced computer technology facilitated the development of the inaugural electromechanical electronic gaming machine featuring bonus games, multiple paylines, and contemporary virtual reality games. With modern internet access, operators have cultivated an extensive realm of advanced machine gameplay and a substantial demographic of EGM players. In contrast, online game producers are primarily constrained by their creativity. The inaugural video EGM, using a dual-screen setup, was developed in Australia and subsequently adopted by America. The other screen serves to immerse the player in an alternate environment for bonus gameplay. The most evident alteration in the EGM’s design was the substitution of single-colour Light-Emitting Diode (LED) signage with multi-colour Liquid Crystal Display (LCD) touchscreens for the player reward system.

Burton (2009) elucidates that prominent EGM manufacturers have recently developed ergonomically designed EGMs to enhance player comfort during gameplay. The previous iteration of EGM was intended for players who would typically engage in a few games before departing. The contemporary EGM is engineered to ensure player comfort, encouraging prolonged engagement with the machine. Other machines are engineered with seats that enhance the architectural design of the EGM while ensuring optimal comfort for the user during gameplay. While comfort is not the primary objective, the games are more engaging and are compatible with mobile devices. The charging port allows consumers to charge their phones or other USB-powered devices at the machine. Illumination synchronises with gaming experience, music, and auditory effects.

### 2.2 Understanding artificial intelligence unit systems

Artificial intelligence is the tool most commonly used by casino operators to measure punter behaviour. This system forms an integral part of any land-based customer relationship management system.

Artificial intelligence Unit (PTU) is used to reward players who maintain loyalty to the casino operator by continually visiting the casino regularly, therefore players will enroll to obtain a benefit from it and again this device or the tool is used by casino operator to trace and check the performance or the behaviour of the punter from one machine to another. These devices equip casino operators with information about the behaviour of the machine and the punter around the casino floor, or overall player acquisition and retention strategy. Without this device, the casino operator’s manager will have to conduct walkabouts or rely on casino floor staff to determine which machines have been played the most, what their preferences are, and their interest in the casino floor.

McGowan (2021) describes the Monitoring and Control system as the computerised server that monitors and measures the behaviour of the punter on the casino floor. The information is generated by the AI player tracking unit and transmitted to the server, where the casino operator uses it for machine positioning. The Monitoring and Control System (MCS) is a computerised server that monitors all the EGM machines on the legalised operator floor and ensures that they comply with legal requirements. The installed software monitors game performance, winning, significant events and data visualisation. The data visualisation helps the operator monitor events in real-time and see the customer interacting with the machine, as well as how much they are spending. Most of these AI devices are built with a responsible gambling program code of conduct in mind. However, the information is only managed by operators who participate in responsible gambling programmes.

According to Velotta (2022), the Artificial Intelligence System (PTS) is an automated system used by an operator to generate significant events for marketing purposes, monitor players, and offer rewards to punters who maintain loyalty to the business by continuously playing the EGM. This operation is performed by enrolling in the operator’s database to obtain benefits. Moreover, this operation depends on the individual operator, and again, it depends on what is permitted in the jurisdiction where the operator is regulated. The lax regulations or rules leave the operator to design the system that will induce its customers to play more on the EGM.

The introduction of AI systems in the land-based environment has revolutionised the gaming community, where operators worldwide rely on the data that AI systems collect and analyse. The system is used to enhance gaming experiences. Most of the previous studies have shown that the AI system was introduced after the decline of Hopper Coin.

The functions of a basic AI system in casino operators include the following functions (a) total playing time or duration (b) turnover or returned to the punter (RTP) (c) theoretical loss of any game (d) game themes (e) Gross Gaming Revenue (GGR).

The AI system has evolved into a flexible platform with features tailored to players, resulting in a unique and interactive gaming experience. After players enter their player cards into the tracking system, the system automatically records and links all of their gaming activities to their loyalty accounts. The system can log users in and track their gaming progress, but it may also offer additional games or incentive schemes to boost motivation further. In response to a player’s wagers or loyalty, the casino may credit their account with rewards, such as free play, bonus points, or even access to exclusive membership benefits, including the hotel’s facilities. Casino operators will also benefit from this improved contact, as it will enable them to learn about each player’s unique preferences and the impact of their marketing initiatives on individual players.

To assess the severity of problem gambling, all surveys utilised the nine-item PGSI (Ferris and Wynne, 2001). On a four-point scale, respondents indicated the frequency with which each item pertained to them within the past 12 months: 0: never, 1: sometimes, 2: most of the time, 3: practically always. Ratings vary from 0 to 27, with higher ratings indicating increased problem severity. PGSI scores can categorise individuals as non-problem gamblers (score of 0), low-risk gamblers (scores of 1 or 2), moderate-risk gamblers (scores ranging from 3 to 7), or problem gamblers (scores of 8 or above). Despite the PGSI typically exhibiting robust psychometric properties (Ferris and Wynne, 2001; Turner and Horbay, 2004; Neal et al., 2005), a straightforward scoring adjustment has been suggested, wherein moderate risk gambling is characterized by scores ranging from 5 to 7, due to findings indicating inadequate discriminant validity between the low and moderate risk categories (Turner and Horbay, 2004). The PGSI has been established as the preferred measurement instrument for population-level research in Australia (Neal et al., 2005).

Ferris and Wynne (2001) as well as Sacco and Jeong (2025) affirmed that the Problem Gambling Severity Index (PGSI) is a technique utilised by numerous researchers to ascertain the veracity of whether a gambler has attained the status of an irresponsible or pathological gambler. There is a mechanism in place to monitor all gaming establishments. To ensure the EGM on the property is operational, the operator may utilise this system. Additionally, the same technology tracks which slot machines each player prefers to use while at the casino. An AI system can monitor a new player’s performance on the slot machines and notify the operator if they show signs of being a loyal customer. With its exceptional performance, you can extract data on a daily, weekly, or monthly basis, and it continuously gathers data.

### 2.3 Consequences of irresponsible gambling

Cabot and Pindell (2025) advocate the view that gaming is one of the economic drivers. However, the government faces many problems associated with gambling, such as money laundering, alcohol and drug abuse. In a non-secular nation or a religiously dominant country, this question is easily answered. Several religious nations condemn any form of gambling activity.

Gambling serves as a recreational activity and also provides job creation and generates revenue for the government through taxes and levies. However, Markham et al. (2016) found a correlation between gambling related losses and harm, which implies that irresponsible gambling could lead to harm.

An individual with a gambling issue may display a multitude of symptoms. Friends, coworkers, and relatives may notice that the person is distant, sleepy most of the time, always seeking financial aid, depressed, anxious, or worried about nothing, without fail, leaving the house to visit a licensed gambling establishment or even partake in illicit gaming.

Harris (2019) indicated that most of the problem gamblers who were treated or requested gambling counselling are slot machine players, with 53% compared to other gambling modes. However, a recent study by the National Gambling Board reveals the decline of slot machine playing, but it still has an impact on the gambling industry.

National Gambling Board (2022) shows that gambling on Electronic Gaming machines is a significant contributor to the Casino industry’s profits. However, the profit margin may vary across operators; some operators have gained even higher profits on slot machine gambling. Harris (2019) indicates that there are problem gamblers for every 10,000 persons in three of South Africa’s Provinces. Gauteng Province tops the list, although this information does not necessarily mean that Gauteng has the highest number of irresponsible gamblers. It may be because there are more people in Gauteng, or it may be due to poor prevention programmes.

The statistics in Table 1 reveal that problem gamblers are spread across the South African Provinces, which constitutes a problem to the government and society. If the responsible gambling programme is effective, it will bring better awareness to the public. The resources and effort must be channeled to all South African Provinces, especially the Gauteng Province, due to the higher number of people per capita who gamble.

**Table 1:** The referral of gambling problems per capita.

Characteristics of Selected Provinces in South Africa	**Selected Provinces**					Average of **5** provinces
	Eastern Cape	Free State	Gauteng	KwaZulu-Natal	**Western Cape**	
Total Population	6,562,053	2,745,590	12,272,263	10,267,300	5,822,734	37,669,941
>18	3,938,520	1,792,205	8,849,052	6,310,572	4,083,309	24,973,659
No problem, Gambling Referrals	850	373	6,225	2,138	1,954	11,540
% of >18 years referred to Gambling Problem	0.0218%	0.0208%	0.0703%	0.0339%	0.0479%	0.0462%
Out of every 10,000 persons over 18	2	2	7	3	5	5

**Source:** Eastern Cape Gambling and Betting Board and South African Responsible Gambling Foundation (2017).

Patrick (2019) argues that land-based slot machine gambling and online (virtual) slot machine gambling. However, they differ in their offerings, but they both offer amusement and entertainment to players. Both machines have similar features and popular themes, featuring bright colours, flickering lights, and enticing sounds. These machines were designed to lure potential gamers to the gaming area. Braschke (2022) stated that a well-known casino operator in Australia was fined heavily after failing to adhere to a responsible gambling code of conduct. The operator also showed a lack of willingness to help prevent the harm that can be associated with gambling for long hours without a break.

Auer and Griffiths and Wood (2008) as well as Auer and Griffiths (2013) describe the responsibility of limit setting as a pre-committed tool that allows the punter to set the loss limit (which could be a day, week, or Month) or the amount of money they are willing to lose at the electronic gambling machine. This tool allows the punter to set the limit before engaging with the EGM. The limit-setting practice is viewed by scholars and members of the gambling society as a method of putting informed decisions at the heart of a responsible gambling program.

Auer and Griffiths et al. (2013) explain the options for spending limits available to the responsible gambling Operator. This spending limit helps the punter preset the limit before engaging in gameplay, such as gameplay limits, deposit limits, and Loss Limits. This option is voluntary in some jurisdictions, while in others it is mandatory. Griffiths et al. (2009) noted that the pre-commitment tool (fixed limits set by the operator) is seen as a positive development by the punter. It does not necessarily encourage gamblers to take sole responsibility for managing and monitoring how much money/time they spend in the gambling environment. Water (2022) proposed the win limit in a recent study. The win limit is the amount of money a punter can win in the EGM. This feature was tested with several punters on a simulated electronic gambling machine, and it was found that a self-imposed win limit would lead to the punter playing more and thereby reducing the operator’s profit.

Binesh et al. (2026) indicated that the future of gambling will be driven by AI. The outcome of the study revealed that the future gambling activities will be influenced significantly by human-AI collaboration, policy and regulatory changes, AI modelling for predictive analytics, gaming system and player interaction. Binesh and Ghaharian (2025) also stressed that the introduction of AI into gambling activities will require AI ethics for compliance and to mitigate the evolving risks and that gambling regulators and operators must be aware of this.

Murch et al. (2024) stressed that AI-based gambling harm detection systems could enable automated intervention and treatment referral for high risk or irresponsible gamblers.

Murch et al. (2025) indicated that many gambling detection system are based on behavioural patterns hence, the introduction of socio-demographic factors into the modelling is important as this will enable a personalized harm preventive approach.

Sacco and Jeong (2025) found that irresponsible or problem gambling is more prevalent was more among the loyalty program players than typical in a population sample suggesting the need for more lottery loyalty programs for preventing gambling addiction.

### 2.4 Application of artificial intelligence to gambling

Gambling is now paramount amongst the youth and has rendered many unproductive. Gambling is considered responsible when people do not depend on it as a primary source of income or commit their lifetime earnings to it, and when they do not engage in it frequently. However, in South Africa, for people, especially the youth, gambling has become a source of problems because of its adverse effects on the lives of people, including their family, relationships, work, and studies. Some factors responsible for irresponsible gambling include gender and social norms. For instance, men were more likely to gamble than women, and people are more prone to gambling in a society that accepts it as a norm (South African Responsible Gambling Foundation 2024).

Irresponsible gambling has many consequences, such as:

- Bankruptcy: it can lead to severe financial problems, such as the loss of valuable assets.
- Relationship problems: it can affect relationships amongst family and friends, leading to loss of trust, child abuse, domestic violence, etc.
- Mental health crisis: gambling can lead to mental health issues such as depression, anxiety and other mood disorders.
- Substance abuse: gambling can lead to other risky behaviours, such as alcoholism and the use of prohibited substances.
- Crime: gambling can promote social vices, such as theft and fraud.
- Suicide attempts: gambling can lead to suicidal attempts or thoughts.
- Under productivity: due to mental health issues and cumulative effects of the above-mentioned factors, irresponsible gamblers may be less productive in their workplace, society, etc.

This study, therefore, proposes an artificial intelligence-based system that can be used to classify individuals as either responsible or irresponsible gamblers. This is to have fun, rather than one that puts a person in a financially, emotionally, or otherwise challenging situation.

The proposed AI system will be able to do the following:

- Set a limit before gamblers walk into a casino or play a game.
- Identify addicted gamblers and bring to light warning signs of irresponsible gambling.
- Ensure gambling is done for limited amounts of time, both in frequency and duration.
- Ensure gambling has both predetermined and acceptable limits for losses.
- Ensure operators comply with the Codes of Conduct of responsible gambling.

There is a dearth of literature on the design of EGM, which can bring information on how EGM has been designed, as well as the types of games and the way the equipment contributes to problem gambling or leads to gambling disorder. This information will reveal if the manufacturer deliberately concealed information related to the potential and harmful addictive nature of the device. The effect and the evidence related to virtual EGM causing gambling disorder are still under review by different bodies of researchers.

Furthermore, there is a lack of sufficient literature on the application of artificial intelligence-based models for promoting responsible gambling. This is the central focal point of this study: to propose effective strategies that can result in harm minimisation during gambling.

The outcome of the literature survey indicates that in a traditional land-based gambling environment, it is well known that among loyalty card reward members, 20% of loyalty card holders account for 80% of the operators’ revenue.

Based on numerous studies, it was concluded that more studies and improvements need to be made through behavioural gambling, such as gambling under limited or specific time and taking a break when playing. Betting more than a specified amount on the machine, gambling through mealtime or withdrawing cash from an Automatic Teller Machine (ATM) must be discouraged. In the studies reviewed, there were no interventions from the staff to interrupt the gambler from taking a break, nor was there any insight into the machine design that stops problem gamblers.

## 3 Methodology

This section presents the methodology employed and details the procedural steps involved in developing the artificial intelligence-based model for responsible gambling.

### 3.1 Research design

To assemble this literature review, I conducted a meticulous search of the internet for articles on responsible gambling, as well as electronic databases, papers, and journals. The causal explanation argument posits that electronic gambling machines affect problem gamblers; therefore, the researcher selected a case study that aligns with the exploratory research methodology.

**Figure 1:**
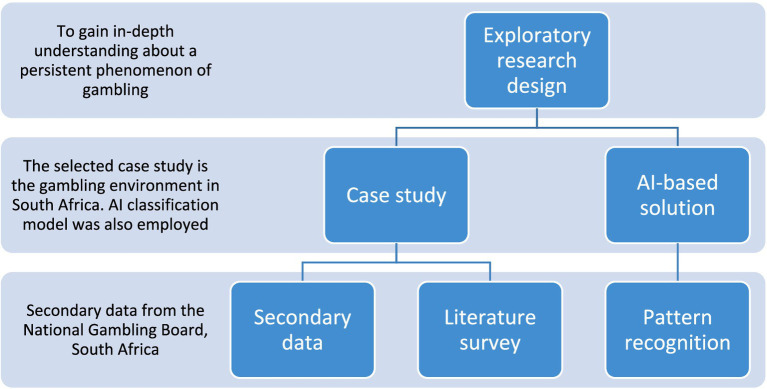
The research design.

The goal of this exploratory study is to learn as much as possible about gambling as a continuous occurrence. Consequently, the inductive approach is employed in the exploratory phase to explain the phenomenon and identify the underlying general principles. According to Zainal (2007), exploratory research often involves gathering and organising secondary data for synthesis. Consequently, this study relied on secondary data collection and analysis. Using secondary data sourced from the South African Responsible Gambling Foundation, South Africa, this paper examines the gambling environment in South Africa as a case study. The study’s research plan is shown in Figure 1.

Thus, this study combines an explanatory research design and machine learning model development, validated using a secondary dataset.

The choice of artificial intelligence (AI) model for promoting responsible gambling stems from the fact that AI models are capable of monitoring real-time behaviour, thereby allowing EGM operators to dynamically assess risk based on the behaviour of the player, such as the player’s interactions with the machine and frequency of betting, amongst others. Traditional strategies to enhance responsible gambling (such as static warnings and time limits) lack real-time adaptability, flexibility and responsiveness that AI systems can provide. Auer and Griffiths (2013) note that AI systems can continuously learn from user interactions and update their predictions in real-time, making them ideal for gambling environments.

Furthermore, AI solutions can provide early detection of irresponsible gambling patterns through the analysis of historical and real-time behavioural datasets to identify patterns before they become visible to human observers, thereby making it more proactive compared to traditional approaches. Furthermore, it can provide personalised interventions or controls by ensuring that warning signals and feedback are tailored to individual players based on their unique behavioural profiles, reducing the likelihood of alienating or irritating casual players. Auer and Griffiths (2023), stated that personalised controls are effective risk mitigation strategies in gambling than the traditional approach.

In addition, AI models boast of scalability and efficiency when compared to traditional approaches. This is because AI solutions can monitor large datasets and a vast number of EGMs with high accuracy, making them highly scalable across large casinos or online platforms in a cost- and time-effective manner (Harris, 2017).

Thus, the integration of an AI solution into EGM is justified by its capability for real-time monitoring, analysis and controls in player behaviour, thereby offering a proactive, personalised, and scalable solution to promote responsible gambling. AI solutions are data-driven and require ethical procedures for a robust outcome.

### 3.2 Data collection and analysis

Secondary anonymised datasets on EGM and gambling in South Africa were obtained from the South African Responsible Gambling Foundation (2024) and the National Gambling Board (2022). The dataset comprises of 342 observations across four player categories namely Marron, Gold, Silver and Platinum. The three gambling frequencies (weekly, biweekly and monthly), session times, player significant events per visit, machine usage, bet sizes, as well as the ratio of wins to losses are presented in Table 2. The problem is defined as a multi-class classification problem to categorise responsible, intermediate and irresponsible (Problem gamblers) based on gambling frequencies.

**Table 2:** Player significant event per visit.

Programme	1–5 times a month	1–5 times in 2 weeks	1–5 times a week	Total	Frequency (%)
Marron	50	60	30	140	40.90
Gold	25	70	38	133	38.90
Silver	28	18	8	54	15.80
Platinum	10	4	1	15	4.40
Total per visit	113	152	77	342	100

Ethical compliance with the Protection of Personal Information Act (POPIA), and other relevant data protection laws was ensured by anonymising the dataset.

The Microsoft Excel software was used to arrange the data of players’ significant events. The pie charts and a bar graph show the spending pattern of supposed problem gamblers. Statistics provides a summary of data that includes measures of average and variability of information. The graphs, such as scatter plots and frequency tables, visualise data to check any trends and information. Table 2 displays the number of visits per player.

The visit illustrated above does not refer to the amount spent, but rather the number of visits to the operator. Some punters gamble responsibly within their budget.

### 3.3 Procedural steps in the development of an artificial intelligence-based model for responsible gambling

Artificial intelligence, specifically the machine-learning pattern recognition and classification model, is a learning approach that learns from a trained dataset, allowing decisions or predictions to be made based on the established relationships in the dataset’s historical data. Gambling is an unpredictable activity; therefore, it requires the use of a versatile algorithm that can analyse the interrelationship between the factors responsible for irresponsible gambling and identify anomalies in the dataset to establish a trend or pattern. Machine learning was preferred and selected for use in this study because it can detect the activities of gamblers and enable future trends or predictions (Akinbowale et al., 2023).

The following procedure steps were undertaken during the implementation of the artificial intelligence-based model.

#### 3.3.1 Data pre-processing in the MATLAB environment

The goal is to clean and prepare the gambling dataset for modelling. The dataset comprises 21 matrices with 7,200 gamblers. It is characterised by 15 binary and six continuous attributes of irresponsible gamblers, namely: age, gender, gambling duration, frequency, set limits, and gambling environment. The target, however, comprises a 3×7200 matrix of 7,200 associated class vectors. This defines the type of the three classes each input is assigned to. The classes are denoted by a 1 in row 1, 2 or 3.

Where 1 represents responsible or normal gambling, two denotes intermediate, and 3 represents irresponsible or abnormal gambling and [X, T] represents the input and target variables, respectively.

The dataset was stored in the CSV format in the Excel and imported into the MATLAB 2017a environment using the code below:data = readtable(‘gambling_data.csv’); The missing values were identified and removed using the code below:
summary(data); % Shows NaNs and missing data.
data = rmmissing(data); % Remove rows with NaNs.


This stage was followed by the normalisation of the numerical features, such as the frequency of machine usage and session time as well as the conversion of the categorical variables into numerical form using the respective codes below:dataNorm = normalize(data(:, {‘Frequency’,'SessionTime’}));
data. Gender = grp2idx(categorical(data. Gender));


A pattern recognition model was developed to identify the trend of gambling activities in South Africa which classifies gamblers into three categories: responsible, intermediate, and irresponsible. The implementation of the classification model was done in MATLAB 2017a. Responsible gambling is considered to comply with the ethical code of conduct of gambling activities, while irresponsible gambling breaches the ethical code of conduct of gambling activities. Intermediate gamblers are people who breach some of the ethical code of conduct of gambling activities and are gradually transitioning into irresponsible gambling. Early detection through an artificial intelligence-based solution can send a strong warning to operators to monitor these individuals and, where possible, warn or prevent them.

#### 3.3.2 Feature engineering

The essence of this phase is to extract vital characteristics from the dataset that can assist the model in differentiating between responsible and non-risky behaviour and irresponsible and risky behaviour. The behavioural features of the gamblers considered include time played by the gambler per session (session duration), escalation of gambling trends, win/loss patterns, frequency of plays (number of sessions played per day/week), as well as near-miss outcomes. The MATLAB environment uses functions such as “fscnca,” “relief,” and “sequentialfs” to automatically select and rank the identified features automatically.

#### 3.3.3 Hyperparameter tuning

The goal of hyperparameter tuning is to configure and optimise the machine learning algorithm settings (hyperparameters) to enhance the model’s performance. For the Scaled Conjugate Gradient backpropagation algorithm, hyperparameter tuning ensures high accuracy, better generalisation, and rapid convergence.

Table 3 presents the division of the dataset. The data division was performed randomly, as shown in Table 3. The scaled conjugate gradient backpropagation (train score) was used as the training algorithm, and was evaluated using the cross entropy.

**Table 3:** Division of the dataset.

Type of dataset	Percentage (%)	Number of samples
Training	70	5,040
Validation	15	1,080
Testing	15	1,080
Total		7,200

The larger the dataset, the more accurate the model and the classification assignment, and vice versa.

Table 4 presents the parameters for the classification model.

**Table 4:** The parameters for the classification model.

Parameter	Multi-class
Optimiser/algorithm	Scaled conjugate gradient back propagation
Learning rate	0.01
Regularisation parameter	0.01
Input	21
Hidden nodes for the neuron layer	10
Hidden nodes for the output layer	3
Epoch	107
Loss function	Cross entropy
Gate activation function	Sigmoid
Gradient threshold	1

The number of neurons in the hidden layer (10) controls the ability of the model to learn and capture complex pattern while the number of neurons in the output layer (3) was selected based on the three possible outcomes of classification: responsible, irresponsible and intermediate gambling. The learning goal was to achieve minimum performance error using the Mean Square Error (MSE) as the error metric. The epoch is the maximum number of training iterations conducted. A learning rate of 0.01 was selected to balance speed of convergence and effective learning rat as higher values can speed up the rate of learning but cause instability. The regularisation parameter value of 0.01 was also selected to prevent overfitting by penalizing large weights. The choice of the value was informed by the moderate nature of the datasets characterised with some noise. The MATLAB environment provides an automatic hyperparameter optimisation using the Bayesian optimization and this is done via the feature “fitcnet” which supports automated hyperparameter optimisation. It is necessary to tune the hyperparameters to prevent overfitting or underfitting due to incorrect choice of the hyperparameters. The selected algorithm scaled conjugate gradient backpropagation is sensitive to the size of network and the performance goals. However, when the performance goal is outside the optimum range, it may increase the number of iterations. The training plot was employed to visualise the progress of the training while the confusion matrix was employed to visualise the performance of the classification mode.

The choice of the NN model was informed by the fact that it can capture hidden, complex and non-linear patterns such as association or correlations among the gambling patterns and behaviours. It can also capture the interactions between the variables automatically (Haykin, 2009). Gambling activity is dynamic, pattern based and can be influenced by various factor hence, NN is suitable for behaviour prediction and user multi-classification and pattern recognition.

The hidden layer in a neural network is important in capturing complex nonlinear patterns within the input data. In this study, 10 neurons was selected in the hidden layer to prevent possible underfitting or overfitting as lesser number of neurons may result in underfitting thereby hindering the model’s ability to learn complex pattern. Conversely, the selection of too many neurons may result in overfitting; a situation whereby the model captures irrelevant features such as noise due to excessive learning thereby reducing the performance of the model. Haykin (2009) stated that a *good starting point for the selection of the number of hidden neurons is between the size of the input (in this case 21) and the size of the output layer (in this case 3)*. Thus, the selection of 10 neurons in the hidden layer balances adequate learning capacity and generalisation with optimum computational time and cost.

The number of neurons in the output layer (3) is a reflection of the number of classes to be predicted. In this classification task, the dataset is classified into three distinct classes namely: normal/responsible/risk-free gambler, intermediate gamblers and irresponsible or problem gamblers. Bishop (2006) *stated that in pattern recognition assignment, the number of output neurons should be equal to the number of classes, with the use of the softmax activation to model class probabilities*.

#### 3.3.4 Addressing class imbalance and overfitting

In gambling datasets, challenges such as class imbalance may occur whereby there are fewer intermediate or problem gamblers compared to the number of responsible gamblers or vice versa. This may result in bias toward the majority class thus hindering the model’s ability to behaviours attributed to classes with lesser dataset. To overcome this challenge, three approaches were used in this study. First, the loss function was adjusted by assigning higher cost to misclassifying minority (in this case the problem and the intermediate gambler). Secondly, the stratified data splitting was employed to ensure that each of the classes is proportionally represented in the training, validation, and test sets.

Thirdly, the dataset was increased from 342 samples to 7,200 samples using the Synthetic Minority Oversampling Technique (SMOTE). This technique generates synthetic samples for minority classes via interpolation between existing data points, thus, improving the class balance without duplication of samples (Chawla et al., 2002; He and Garcia, 2009). The resampling process was done to ensure a fair representation of all the classes to reduce bias and enhance generalization.

Lastly, beyond accuracy, other evaluation metrics such as precision, recall, F1-score, and confusion matrix were used to identify and validate that minority classes.

Another challenge frequently encountered in classification task is overfitting. This occurs when the model memorises the training data but fails to generalize to new data, which may likely occur in a moderate or noisy gambling datasets. To mitigate this, the training in the MATLAB environment automatically stops training once the validation error increases for max_fail consecutive iterations (6). Thus, to ensure the accuracy of classification, the model was trained split training, validation, and testing dataset (70%:15%:15% respectively). Furthermore, a penalty was added to large weights to reduce model complexity and the use of 5-fold cross-validation ensures that model performance is evaluated across multiple data subsets.

### 3.4 Measurement of gambling behaviour

Indicators of irresponsible gamblers are frequent gambling, gambling to make money, ignoring the affordability of their gambling activities, risks of losing money they cannot afford, difficulty in setting a limit, and always chasing losses by gambling more (Slabczynski, 2023).

Indicators of responsible gambling are gambling for entertainment, always taking breaks during gambling activities, setting a limit, gambling on affordability and budget, and knowing when to quit. Another indicator is the frequency of gambling. A regular gambler wagers more than three times per week. Wagering. In this specific research, frequency was assessed solely on EGM activity, eliminating all gambling modalities. In the past 12 months, how frequently each week, month, or year have you engaged in gambling activity In the ACT, the frequency items were generally phrased as follows: In the past 12 months, how many times each week, month, or year have you engaged in gambling activity In both jurisdictions, frequency was provided separately for various modalities.

### 3.5 Implemented of the AI model

The Machine Learning (ML) technique was employed on the dataset to identify irresponsible gamblers. Moreover, the ML technique offers an overview of the links and trends within the dataset, facilitating the identification, monitoring, and prevention of reckless gambling.

Specifically, Neural Network (NN) was employed consisting of a two-layer feed-forward architecture and SoftMax was employed to classify the dataset into three categories: responsible, intermediate, and irresponsible gambling. The confusion matrix was used to assess the proportions of accurate and erroneous classifications. The training dataset was utilised to calibrate the model, enabling it to learn from the input data for accurate categorisation. Additionally, the validation test dataset is employed to optimise model parameters for enhanced performance and to assess network generalisation. The process ceases when there is no enhancement in network generalisation, utilising the test dataset to assess the classifier model’s performance.

The program used for the execution of the classification assignment is presented as follows:[x,t] = gambling dataset;
net = patternnet(10);
net = train(net,x,t);
view(net).
y = net(x);
plotconfusion(t,y).


### 3.6 Evaluation of the developed model

The training of the dataset stops when there is no improvement in the generalisation, which is indicated by an increase in the cross-entropy error of the validation sample. It is necessary to minimise the value of the cross-entropy. The lower the values of the cross-entropy, the better the classification assignment, and vice versa. A zero value of cross-validation implies that there is no error. The percentage error indicates the number of misclassified samples, while a value of zero implies perfect classification.

The trained model was also evaluated to determine its accuracy in classifying the dataset into responsible, intermediate, and irresponsible gambling using other evaluation criteria, such as accuracy, F1 score, recall, and precision, which were calculated using Equations 1–4, respectively.

$$\text{Accuracy}=\frac{\mathrm{TP}+\mathrm{TN}}{\mathrm{TP}+\mathrm{FP}+\mathrm{TN}+\mathrm{FN}}$$ (1)

$$\text{Precison}=\frac{\mathrm{TP}}{\mathrm{TP}+\mathrm{FP}}$$ (2)

$$\text{Recall}=\frac{\mathrm{TP}}{\mathrm{TP}+\mathrm{FN}}$$ (3)

$$F1\;\text{score}=2.\frac{\text{Precison}*\text{Recall}}{\text{Precison}+\text{Recall}}$$ (4)

Explanation of terms:

- TP (True Positive): the model correctly predicted a positive outcome.
- TN (True Negative): the model correctly predicted a negative outcome.
- FP (False Positive): the model incorrectly predicted a positive outcome.
- FN (False Negative): the model incorrectly predicted a negative outcome.

**Figure 2:**
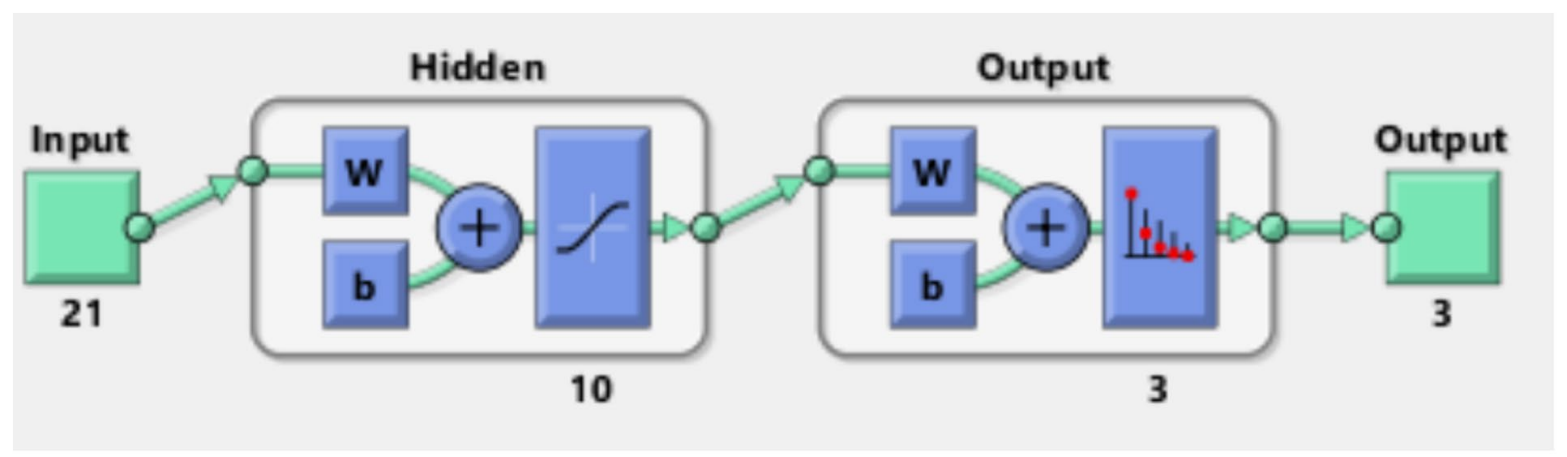
The architecture of the developed classification model.

Figure 2 presents the architecture of the developed classification model, which indicates 21 inputs, 10 hidden neurons and 3 hidden output layers as well as 3 outputs.

The evaluation of the pattern recognition and classification model was done using the cross-entropy and confusion matrix.

### 3.7 Comparison with selected ML models

**Figure 3:**
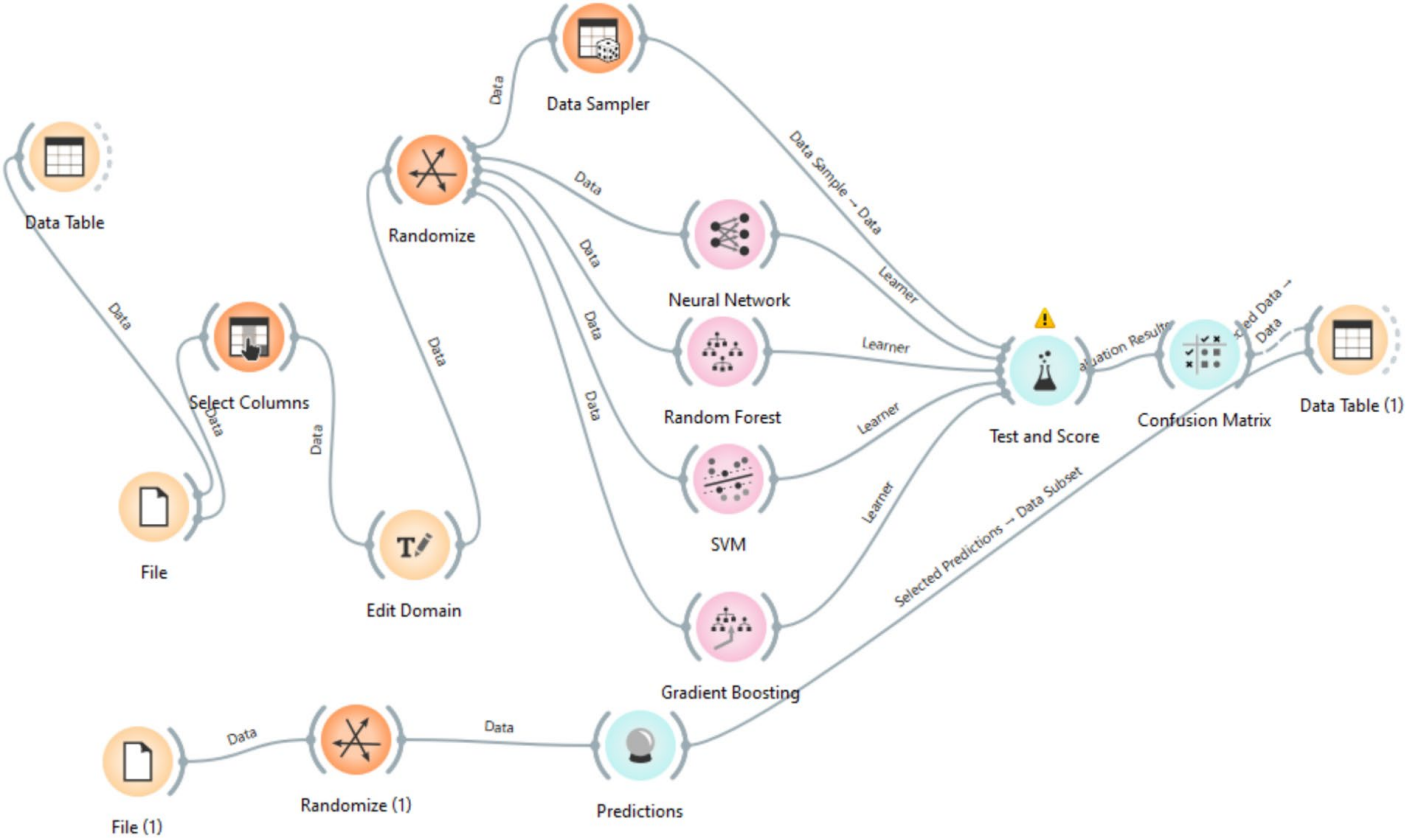
Architecture of the ML models implemented in the Orange environment.

The performance of the NN model developed in the MATLAB environment was compared with the NN, random forest (RF), support vector machine (SVM) and gradient boosting models developed in the Orange environment. The architecture for the models developed in the Orange environment is shown in Figure 3.

The parameters used for the NN in the Orange environment were the same with the ones used in the Orange environment (presented in Table 4) except the scaled conjugate gradient back propagation algorithm was replaced with Adaptive Moment (Adam) optimizer in the Orange environment due to its compatibility.

For the RF models, 10 trees were selected while for the SVM the regression loss epsilon of 0.01 was selected for an iteration limit of 100. For the Gradient Boosting 100 trees were selected at a learning rate of 0.1. The hyperparameters were automatically tuned in the Orange environment.

Figure 3 presents the architecture of the ML models implemented in the Orange environment.

## 4 Results

### 4.1 Statistical analysis of the dataset

Table 5 shows the descriptive statistics of the dataset. The outcome shows that biweekly gambling behaviour dominates the dataset with a frequency of 44% and highest mean of 38 while weekly gambling activity was found to be the lowest gamblers. The mean (38) and variation (66) for biweekly confirm that it is the central behavioural pattern and behaviour of gamblers varies widely across programmes for this period. The high variability implies that various players behave differently within the same frequency. The standard deviation shows that gambler behaviours are more stable for monthly (14.29) and least stable for biweekly (27.68). This shows that gamblers who in gambling monthly are more consistent and predictable than those who participate biweekly.

**Table 5:** Descriptive statistics of the dataset.

Statistic	Monthly	Biweekly	Weekly
Total Frequency	113.00	152.00	77.00
Percent (%)	33.00	44.40	22.50
Mean	28.25	38.00	19.25
Range (Variation)	40.00	66.00	37.00
Variance	204.19	766.00	231.69
Standard Deviation	14.29	27.68	15.22

In terms of programme behavioural patterns, “Marron” peaked at biweekly (60) indicating consistent participation while “Gold” peaked at biweekly (70) and weekly “38” indicating high gambling engagement by frequent gamblers.

The “Silver” programme is moderate for monthly (28) and declines sharply thereafter indicating less consistent or occasional player participation. The “Platinum” programme has low frequency indicating low participation. From the descriptive analysis, it can be inferred that players prefer gambling activities that occurs at moderate frequency (biweekly) compared to the ones that occur at frequency (monthly) or the ones that occurs frequently (weekly). The “Marron” and “Gold” programmes are the primary player base which shows high frequency engagement.

The Chi-square test was also conducted to test for the significance of association between the gambling programme and frequency. The outcome gave a Chi-square (χ^2^) value of 29.37, *p*-value of 0.0000517 at 6 degrees of freedom. Since the *p* < 0.05, the result is statistically significant, indicating that there is a strong association between gambling programme and gambling frequency. This further implies that the behaviour of the players is strongly influenced by the programme they belong to. This may imply that those who subscribe to the “Gold” programme are most liable to increase gambling frequency. Those who subscribe to the Marron programme users show consistent behaviour across all levels which may make them vulnerable or addicted to gambling. Platinum users show very low participation across all frequency categories.

Conclusively, there is a statistically significant relationship between programme type and gambling frequency. While the “Marron” and “Gold” programme dwas found to ominate gambling activities, biweekly gambling was the dominant behaviour pattern.

Furthermore, the Pearson correlation was employed to investigate the linear relationship between the gambling frequencies (variables). The values obtained fell between 0 and 1 indicating that there is a linear relationship between the gambling frequencies (Table 6).

**Table 6:** Pearson correlation coefficient.

Frequency of gambling	Monthly	Biweekly	Weekly
Monthly	1.00	0.63	0.58
Biweekly	0.63	1.00	0.998
Weekly	0.58	0.998	1.00

### 4.2 Results obtained during the training of the dataset using NN model in the MATAB environment

Table 7 presents the training results obtained during the training of the dataset in the MATLAB environment.

**Table 7:** The training results of the NN model in the MATLAB environment.

Parameter	Value	Remarks
Epoch	107 out of 1,000	This implies that the total iteration was 107 out of a possible 1,000. This implies that the model converges easily. Ease of convergence is a positive indicator of a well-developed model, implying that the training process is stable and that the model parameters, such as weights and biases, remain unchanged upon further iteration.
Time	2 s	The model reached 107 iterations in only 2 s, at which point it converged out of 1,000. This also lends credence to the fact that the model converges easily.
Performance error	0.0521	The minimal performance error indicates that the model is adequately trained and suitable for classification activities.
Gradient error	0.00669	The minimal gradient error indicates a high degree of agreement between the actual and predicted datasets, implying that the model has been adequately trained for classification purposes.
Validation check	6	At the validation check of 6, there was no validation failure, which implies that there is no evidence of data overfitting.

**Figure 4:**
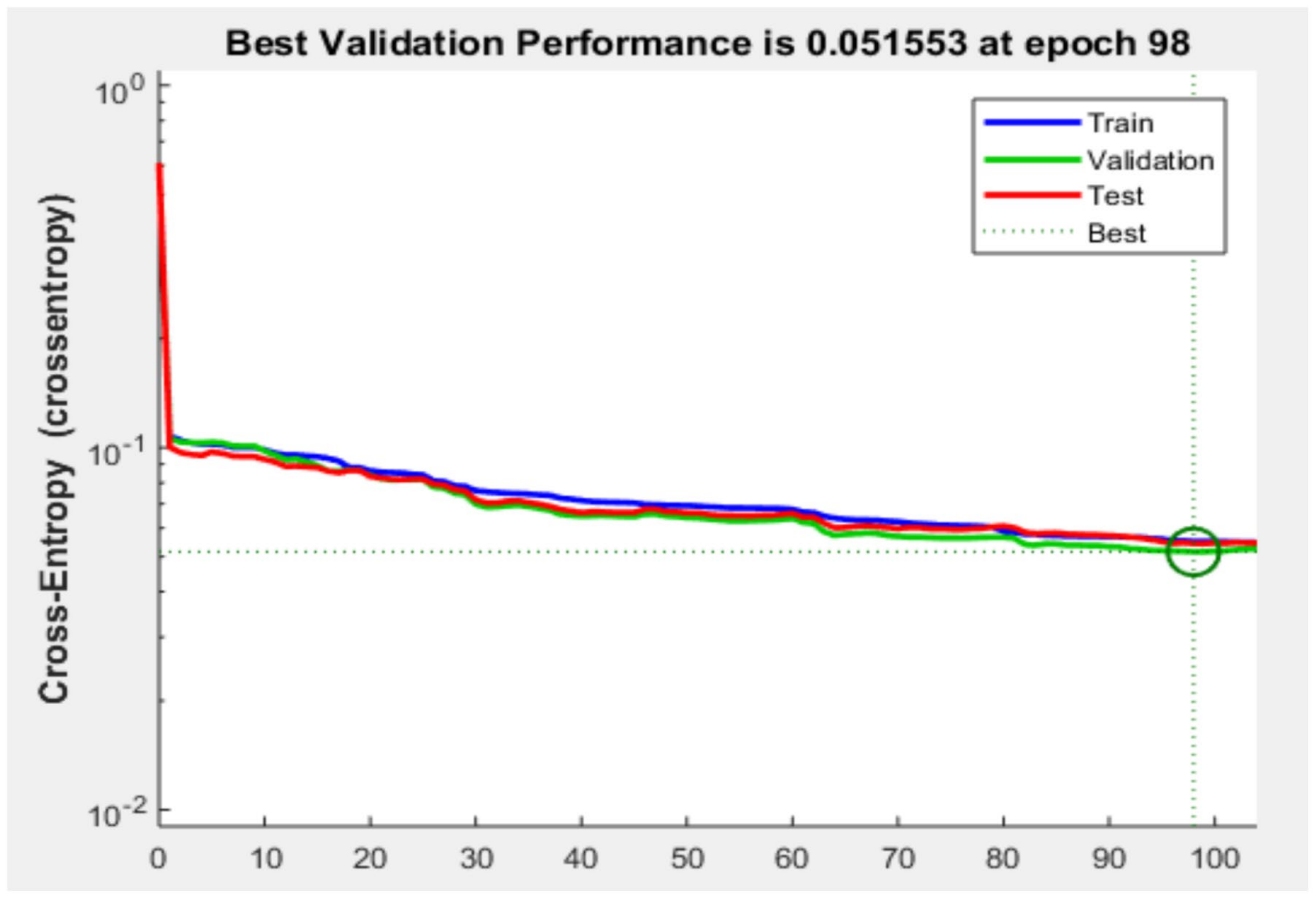
The cross entropy performance goal of the developed NN model in the MATLAB environment.

Figure 4 illustrates the model’s performance, as evaluated using MSE and cross-entropy. The MSE of 0.051553 is negligible, indicating minimal error, and the MSE also observed to decrease as the network training progresses. This implies that the model improves during iterative training. The training stopped at the 107th epoch with a negligible MSE of 0.051553. The epochs refer to the maximum number of iterations performed.

**Figure 5:**
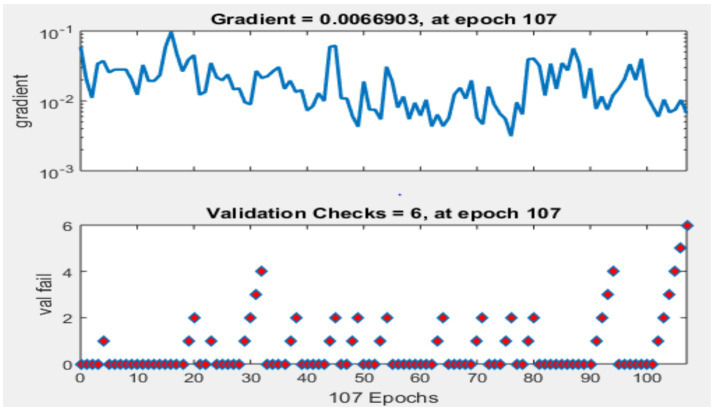
Gradient and validation check plots of the NN model in the MATLAB environment.

Figure 5 presents the gradient and validation check plots during the training of the NN model in the MATLAB environment. The gradient value is 0.0066903 at 107 epochs. The training stopped at the 107th iteration due to observed signs of data overfitting. For validation checks of 6, the best validation performance indicates that there were no validation failures at the 107th iteration. This shows that the model was adequately trained for the classification assignment. After the 107th iteration, evidence of overfitting in the dataset was observed, which may lead to an increase in the MSE. Hence, the model automatically stops once there are no changes in the model parameters upon training to prevent overfitting.

**Figure 6:**
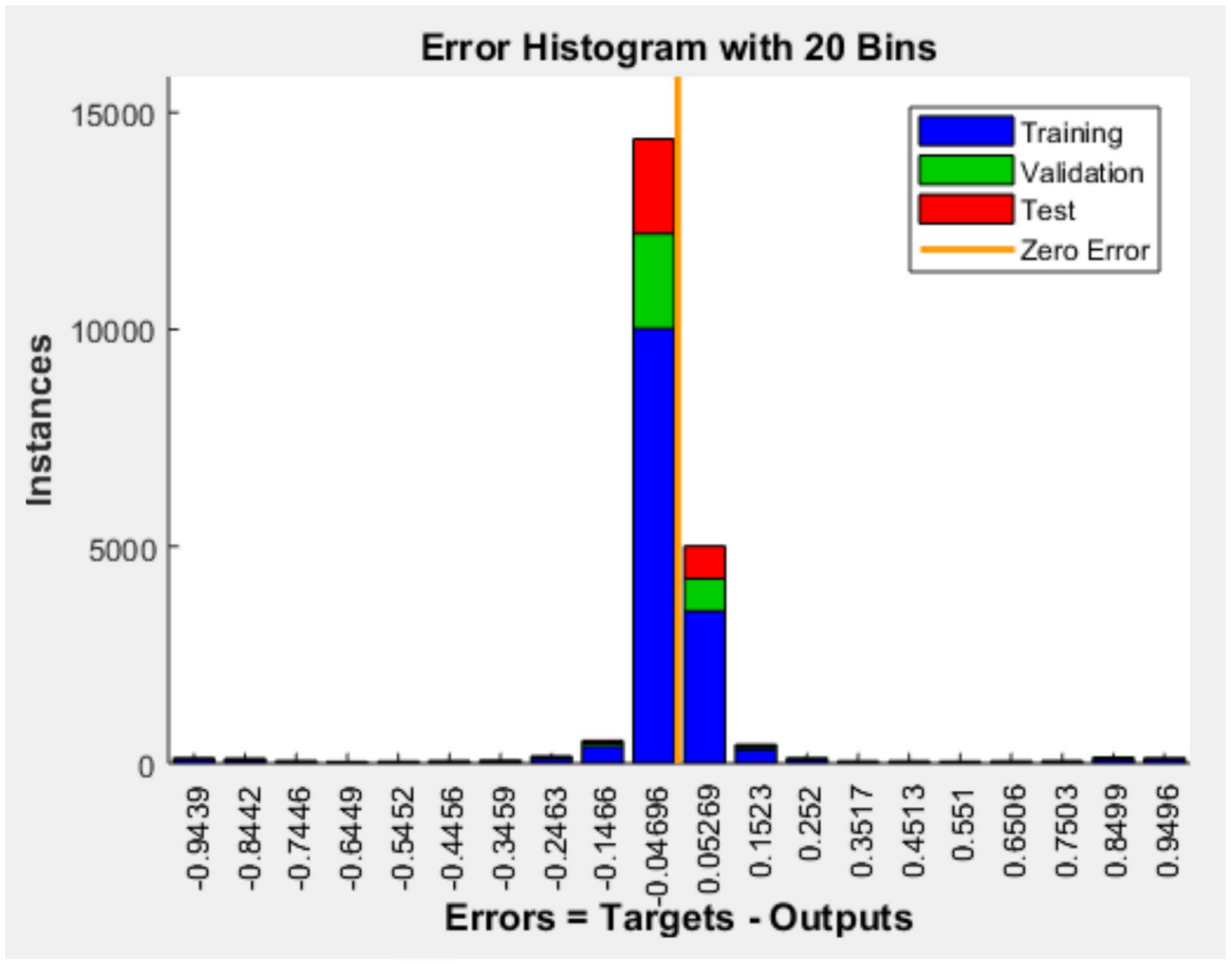
Error histogram.

Figure 6 shows the error histogram plot of the NN model in the MATLAB environment. The error was calculated by subtracting the network’s targets from its outputs. Large values of error imply that the model is not adequately trained and may not be suitable for the classification assignment while a negligible error value indicates that a proper trained model for classification assignment. The figure shows a negligible error values of which implies a significant agreement between the inputs and the output targets. The error ranges from −0.9439 to 0.9496 and the magnitude of error ( E ) was calculated using Equation 5.

$$E=\frac{x_{1}-x_{2}}{n}$$ (5)

Where $x_{1}$ is the lowest error (−0.9439) and $x_{2}$ is the highest error (0.9496) and n is the number of bins (20).

Thus,

$$E=\frac{-0.9439-0.9496}{20}$$

E=−0.094675

**Figure 7:**
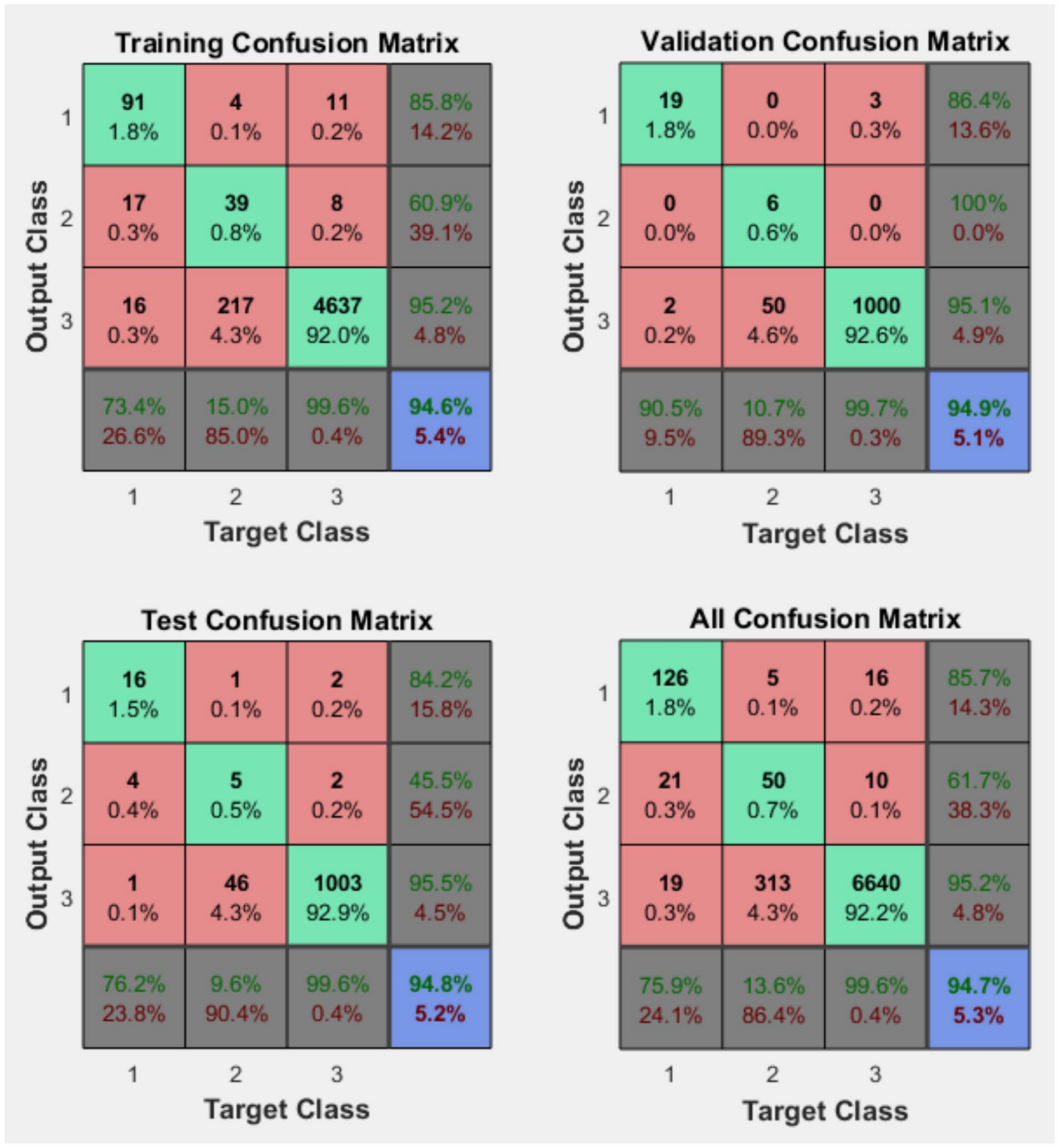
Confusion matrix of the NN model in the MATLAB environment.

Figure 7 illustrates the confusion matrix of the NN model in the MATLAB environment. It displays the performance of the classification model showing the percentages of correct and incorrect classifications. It comprises of four confusion matrices for the training, validation, and test datasets while last matrix represents the overall confusion matrix. The confusion matrix was used to calculate the accuracy, F1 score, recall, and precision of the developed classification model using Equations 1–4, respectively.

The confusion matrix comprises “True Positive” (TP) values, which represent the percentage of correctly classified positive activity. For instance, a TP value is assigned when the model correctly classifies a responsible gambling activity. The “True Negative” (TN), denotes the correct classification of the negative class (when the model correctly classifies an irresponsible gambling activity). On the other hand, a “False Positive” denotes a situation in which the model incorrectly classifies an activity as a positive class when it is negative (for instance, the model classifies an activity as responsible gambling when it is an irresponsible act). Finally, there is a “False Negative” when the model incorrectly predicts the negative class when it is positive (for instance, the model classifies an activity as irresponsible gambling when it is, in fact, a responsible act).

Thus, with the confusion matrix, it becomes easy to visualise the outcome of the classifier algorithm.

For the training process, there was 94.5% correct classification with 5.4% incorrect classification. For the validation process, there are 94.9% correct classifications with 5.1% incorrect classifications. For the testing process, there were 94.8% correct classifications, with 5.2% incorrect classifications.

For the overall confusion matrix, 94.7% of the activities are correctly classified as “responsible gambling,” “intermediate,” and “irresponsible gambling,” while 5.3% of the activities were incorrectly classified. The results show that the percentage of correct classifications is high, with minimal incorrect classifications. This implies that the model is accurate in classifying gambling activities and is also reliable for decision-making purposes.

**Figure 8:**
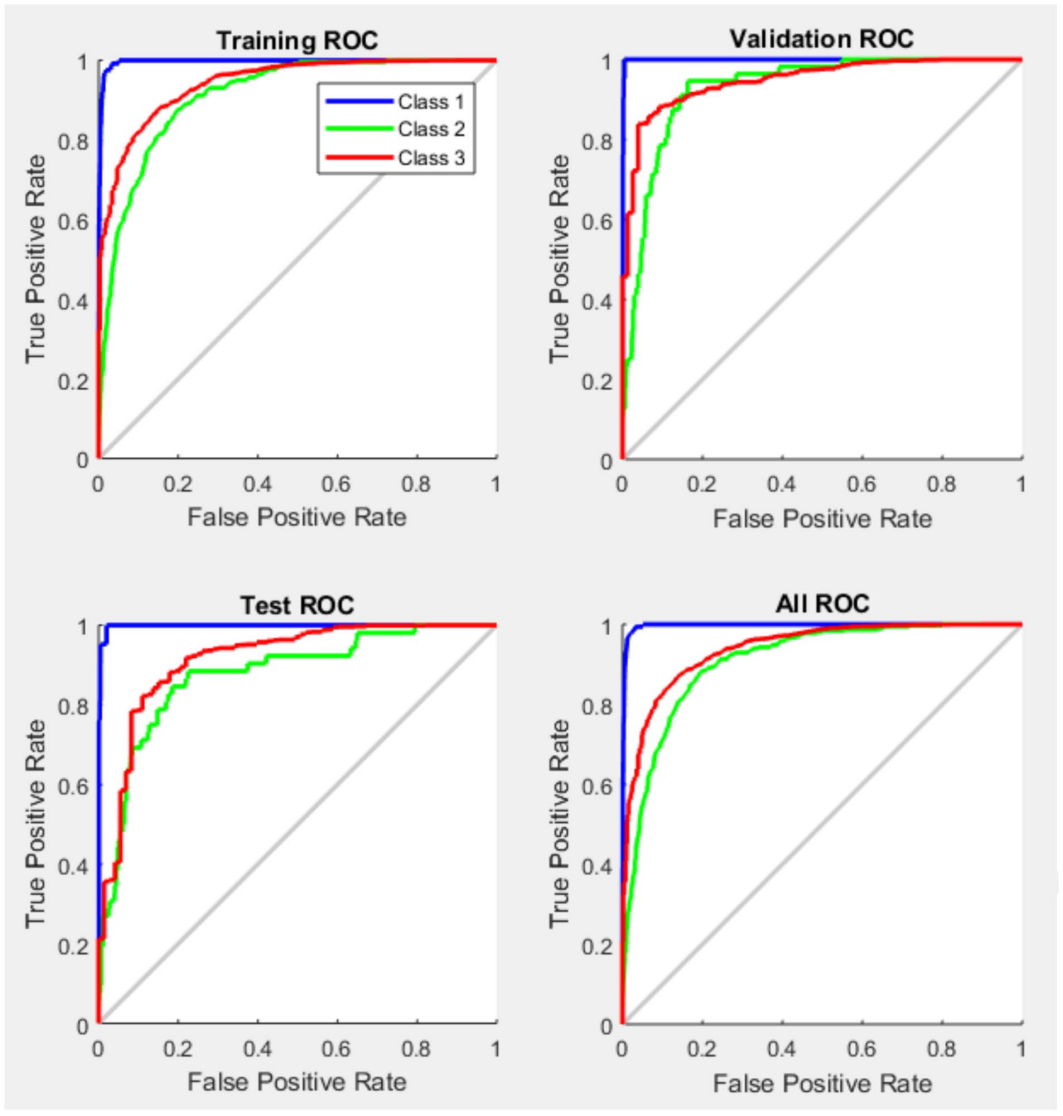
The receiver operating characteristic plot.

The receiver operating characteristic plot of the NN model in the MATLAB environment is shown in Figure 8. This is an indicator of the model’s performance. Class 1 represents “irresponsible gambling,” while class 2 indicates intermediate gambling activities. On the other hand, Class 3 represents irresponsible gambling. The figure illustrates the relationship between the false favourable and accurate favourable rates and the varying threshold of the outputs, which ranges from 0 to 1.

### 4.3 Discussion of results

From the confusion matrix presented in Figure 8, the output and target classes “1,” “2” and “3” denote “responsible gambling,” “intermediate gambling,” and “irresponsible gambling,” respectively. The first row and first column of the overall confusion matrix represent the classification of gambling under “responsible gambling.” In contrast, the second row and second column represent “intermediate gambling” and the third row and third column represent “irresponsible gambling.” Considering the first column, “responsible gambling,” 166 classifications made for “responsible gambling” out of which 126 are correct (true positive). In contrast, 21 gambling activities that should be correctly classified as “responsible gambling” were wrongly classified as “intermediate gambling.” 19 activities that should also be classified as “responsible gambling” were wrongly classified as “irresponsible gambling.” Thus, for responsible gambling, the overall percentage of correct classification was 75.9%, with 24.1% incorrect classifications.

Considering the second column of the overall confusion matrix, at the second column, 368 gambling activities were classified. Five were incorrectly classified as “responsible gambling” instead of “intermediate gambling,” while 50 gambling activities were correctly classified as “intermediate gambling.” On the other hand, 313 activities were wrongly classified as “irresponsible gambling” instead of “intermediate gambling.” Thus, the percentage of correct classification for intermediate gambling was 13.6%, with 86.4% error.

For the third column, which represents “irresponsible gambling,” 6,666 activities were classified. Sixteen activities were wrongly classified as “responsible gambling” instead of “irresponsible gambling” while 100 activities were incorrectly classified as “intermediate gambling” instead of “irresponsible gambling.” 6,640 were correctly classified as “irresponsible gambling.” Thus, the percentage of correct classification for the third column “irresponsible gambling” was 99.6%, with 0.4% error.

Table 8 presents the classification per class.

**Table 8:** Classification analysis per class.

Class	TP	TN	FP	FN
1	126	7,013	40	21
2	50	6,801	318	31
3	6,640	202	26	332
Total	6,816	14,016	384	384

From Table 6, True Positive (TP) was 126 + 50 + 6,640 = 6,816.

True Negative (TN) for class 1, TN₁ = 7,200 − (126 + 40 + 21) = 7,013, for class 2:

TN₂ = 7,200 − (50 + 318 + 31) = 6,801 while for class 3: TN₃ = 7,200 − (6,640 + 26 + 332) = 202. The total was 14,016.

False Positive (FP) equals (166–126) + (368–50) + (6666–6,640) = 384.

False Negative (FN) equals (147–126) + (81–50) + (6972–6,640) = 384.

Hence, using Equations 1–4, the accuracy, precision, recall and F1-score of the classification model are computed and presented in Table 8.

The developed model performed well in terms of average accuracy and recall. This implies that it is reliable and can find all the relevant activities within the dataset and classify them correctly thus overcoming error of omission during classification. However, the average precision and F1-score are slightly close to 1, which implies that the model is prone to false alarms. This was due to the low values of precision and recall for class 2. Despite the use of SMOTE for class balancing, Class 2 still exhibited lower precision and recall. This suggests potential feature overlap, thus, necessitating the need for further feature engineering or model tuning. Future use of hybrid, ensemble or transformer models can also be considered.

Table 9 presents the performance metrics for the evaluation of the developed model.

**Table 9:** Performance metrics of the developed classification model.

Metrics	Class 1	Class 2	Class 3	Average	Remarks
Accuracy	0.947	0.947	0.947	0.947	The value is very close to 1, indicating that the classification model is highly accurate and reliable.
Precision	0.759	0.136	0.996	0.630	It is slightly close to 1, indicating a slight level of agreement between the actual activities and those predicted by the classification model. This implies that there could be some false alarms
Recall	0.857	0.617	0.952	0.809	It is close to 1, which implies that the developed model can find all the relevant activities within the dataset and classify them correctly. This further implies minimal omission error during classification
F1-score	0.805	0.223	0.973	0.667	It slightly to 1, indicating that the classification algorithm can achieve a slight balance between the precision and recall.

The results obtained for the classification model in this study fell within the range of those reported in the literature for the performance metrics. For instance, Shende and Thorat (2020), Awad et al. (2023), and Jony and Arnob (2024) indicate that for a deep learning model, high values of accuracy, precision, recall, and F1-score close to 1 represent a high-performing model.

The outcome is congruent with the research of Sándor and Bakó (2024), who utilised machine learning methodologies to identify and forecast the risky behaviours of gamblers, hence revealing problem gamblers. The study’s results further substantiate that machine learning approaches are effective in forecasting gambling behaviour. The ensemble approaches utilised by Sándor and Bakó (2024) surpassed conventional machine learning techniques, including gradient boosting. Both models effectively identified problem gamblers in real-time. Kozak et al. (2025) demonstrated the identification of individuals exhibiting behavioural traits of problem gamblers using a machine learning model that utilised the CatBoost algorithm. The results of this investigation were also consistent with those of the other study. Mak et al. (2019) and Syed et al. (2020) corroborated that machine learning algorithms are effective for predicting addictive behaviours and gambling habits. Previous research, including that by Percy et al. (2016), has achieved a 35% enhancement in accuracy by applying a Random Forest machine learning model to identify self-excluding gamblers. Ukhov et al. (2021) discovered that the machine learning model effectively recognised parameters, including the utilisation of computers and mobile devices, as well as the daily count of allowed deposits, that contributed to the exclusion of problem gamblers. Finkenwirth et al. (2021) discovered that the random forest machine learning model used for classifying gamblers with and without records of voluntary self-exclusion exhibited commendable performance, achieving an Area Under the Receiver Operating Characteristic curve (AUROC) of 0.75. The current research depended on the observed behavioural patterns of gamblers, including gaming frequency, risk-taking behaviour, and wager amounts, among others. Cerasa et al. (2018) achieved an Area Under Curve (AUC) of 77.3% utilising a Classification and Regression Tree technique, effectively distinguishing between normal and gambling disease.

The results presented in this section indicate that the proposed AI-based solution for minimising harm, tracking players and monitoring gambling activities is feasible. The feasibility is indicated by the performance metrics of the classification model, which fell within the optimal range. The closeness of the accuracy and recall to 1, coupled with the negligible value of mean square error, indicates that the developed classification model is robust and suitable for classification problems. Thus, the findings demonstrate the capability of model in categorising gambling activities into three categories: responsible, intermediate, and irresponsible gambling.

### 4.4 Comparative analysis with selected machine learning models implemented in the MATLAB and Orange environments

Table 10 shows the comparative analysis of selected ML models in the MATLAB and Orange environments. The results obtained indicated that the NN excelled in both the MATLAB and Orange environments compared to other models in the Orange environment. As shown in the table, the performance of the NN model both environments were very close. This further justifies the selection of the NN model.

**Table 10:** Comparative analysis of selected ML models in the MATLAB and Orange environment.

Model and environment	Accuracy	Precision	Recall	F1-score
ANN (MATLAB)	0.947	0.630	0.809	0.667
ANN (Orange)	0.942	0.612	0.833	0.649
SVM (Orange)	0.912	0.576	0.780	0.630
RF (Orange)	0.935	0.515	0.795	0.658
XGBoost (Orange)	0.940	0.520	0.805	0.670

## 5 Development of an artificial intelligence framework for mitigating irresponsible gambling

This section presents the details of the AI framework designed to mitigate irresponsible gambling. It provides practical and procedural steps for implementing the AI-based solution to mitigate irresponsible gambling.

The following are the Procedural Steps for developing an Artificial intelligence framework.

*STEP 1: The first step is to establish the goal of the AI solution*.

The following are the goals of the proposed AI-based solution:

- To set a spending limit before gamblers walk into a casino or play a game.
- Identify addicted gamblers and bring warning signs against intermediate gamblers to prevent irresponsible gambling.
- Ensure gambling is done for limited amounts of time, both in frequency and duration.
- Ensure a break is inserted between the play by using a pop-up animation message.
- Ensuring that gambling has both predetermined and acceptable limits for losses.
- Ensure operators comply with the Codes of Conduct of responsible gambling.

*STEP 2: Collect the dataset and process it.*

To implement the AI-based solution, relevant and recent datasets on gambling operations must be utilised, as the solution is data-driven.

*STEP 3: Identify the risk factors from the dataset and assign risk scores.*

For instance, from the literature survey, some factors are identified as potential red flags known as risk factors for the determination of irresponsible gamblers, including age, gender, gambling duration, frequency, set limits, gambling environment, etc.

Usually, gambling-related harm is commonly assessed via the PGSI (Ferris and Wynne, 2001; Sacco and Jeong, 2025). The PGSI provides a measure of at-risk gambling behaviour during the previous 12-month period. The index is also the measure of the risk score or risk level.

The PGSI predominantly assesses gambling-related harm; however, the term has been broadened to encompass additional gambling-related harm indicators, as well as metrics about quality of life, mental health, and drug use. From the literature survey, several factors have been identified as potential red flags, or risk factors, for identifying irresponsible gamblers. These include age, gender, gambling duration, frequency, set limits, gambling environment, etc.

*STEP 4: Select the AI-based approach.*

Depending on the goal of the AI solution, AI techniques such as deep learning, supervised learning, or unsupervised learning may be suitable for achieving the set target. For this study, the deep neural network approach, specifically the pattern recognition technique, was selected to classify gambling activities into “responsible,” “intermediate” and “irresponsible” gambling operations.

*STEP 5: Implement the selected AI-based solution and evaluate performance.*

Divide the dataset into training, validation, and test sets and select a suitable algorithm to train the dataset, learning the relationships within it to enable it to achieve the predetermined goal. Performance metrics, such as mean squared error, cross-entropy, accuracy, precision, recall, and F1 Score, can be used to evaluate the performance of the AI-based solution.

The intervention and control mechanism of the proposed system will comprise of a multi-tiered intervention system whereby level 1 will provide a visual pop-up reminders (e.g., frequency of playing, time played, money spent) while level 2 will provide session time-outs or cooling-off suggestions and level 3 will offer help resources to the players as well as exclusion options.

*STEP 6: Integrate the proposed AI solution into the existing models.*

This step is necessary to achieve automated real-time tracking, monitoring, detection and prevention of irresponsible gambling activities. The proposed AI-based solution must be integrated into the control and process architecture of the EGM operation to provide timely and real-time intervention or mitigation of irresponsible gambling activities.

For the design of the system’s architecture, it is important to embed the AI module into the EGMs’ software system for real-time behavioural monitoring and control. It is also necessary to ensure low-latency processing that is compatible with existing EGM hardware. Collaborations and partnership with the EGM operators is necessary for effective deployment of the AI-integrated EGMs.

*STEP 7: Test and Review the proposed solution.*

This step is necessary for reviewing the overall performance of the proposed AI-based solution in line with the ethical code of conduct and best standard practices. The proposed solution can be periodically upgraded to keep up with the current dynamics of gamblers and gambling operations. To provide effective review, it is necessary Conduct controlled experiments in a simulated environment to test user response to the AI-driven solutions. The proposed solution will be evaluated in terms of system’s performance, user reactions, and behavioural impact over a fixed period including changes in gambling patterns, user engagement, and incidence of high-risk behaviours will be documented. A Comparative analysis of pre- and post-deployment behavioural analytics should also be conducted to determine the effectiveness of the proposed solution.

Upon successful test and evaluation of the AI-integrated system’s performance, continuous refinement of the AI model using feedback and new data collected during the pilot phase will be necessary. This will be followed by the development of practical guidelines for EGM operators and regulators on implementation of AI for responsible gambling. The guideline will be inclusive of ethical considerations and recommendations for fair and transparent use of AI in gambling environments.

**Figure 9:**
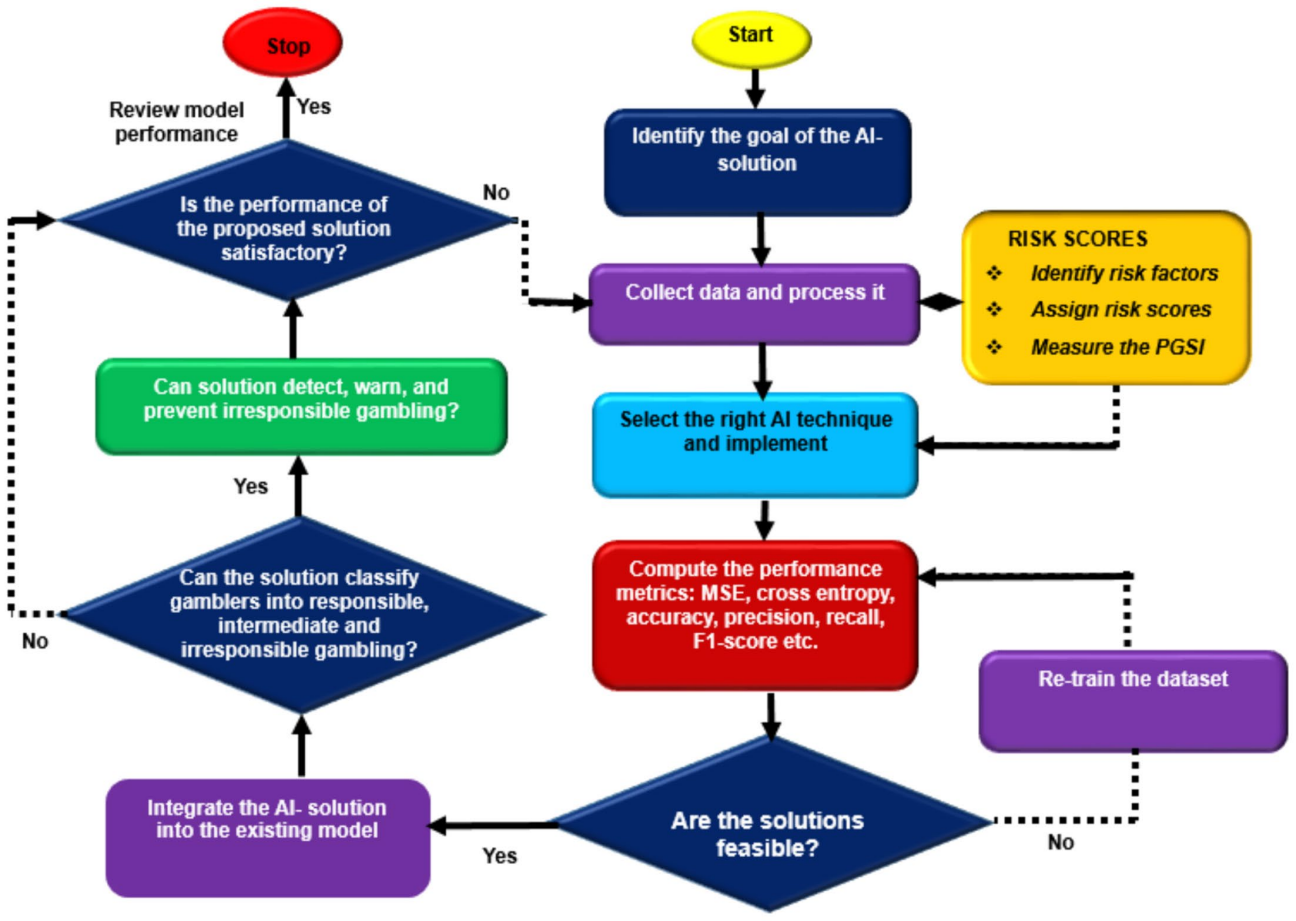
The proposed AI framework for mitigating irresponsible gambling.

These seven significant steps are illustrated in the proposed AI framework, as depicted in Figure 9.

## 6 Conclusion and recommendations

### 6.1 Conclusion

Many casino operators have made minimal efforts to assist gamblers who are grappling with the stigma and shame associated with their addiction, as highlighted in various literature studies. This often results in individuals concealing their struggles from family and friends. Furthermore, responsible gambling codes of conduct and policies frequently lack effective harm minimisation strategies, and self-exclusion criteria are not consistently integrated across different operators. It is essential to recognise that the responsible gambling measures outlined in the Code of Conduct are reactive; they rely on identifying gamblers who are already exhibiting signs of a problem. The classification model developed is not only precise but also highly effective for categorising gambling behaviours. The findings from the classification analysis highlight the impressive capability of the neural network model to distinguish between various types of gambling activities, organising them into three distinct categories: responsible gambling, which reflects healthy play; intermediate gambling, indicating a potential risk level; and irresponsible gambling, which signifies harmful patterns of behaviour. These results underscore the model’s feasibility and its potential application in promoting safer gambling practices. The developed model demonstrated an impressive accuracy of 99.20%, along with a precision of 85.70%. The recall was also 85.70%, resulting in an F1-score of 80.50%. The proximity of these performance metrics to 1, combined with a minimal mean square error, suggests that the classification model is both robust and well-suited for classification tasks.

### 6.2 Policy recommendations

The following are the recommendations that can reduce harm and promote responsible gambling:

1. The implementation of the proposed AI-based solution can detect irresponsible gambling activities and warn or prevent irresponsible behaviours during gambling. This will promote responsible integrity of gambling activities and portray South African citizens as responsible global citizens.
2. The regulators should enforce compliance of the Casino operators to best and standard practices in line with the ethical code of conduct. For instance, regulators should ensure that gambling has both predetermined and acceptable limits for losses.
3. Casino machines may be redesigned to incorporate the AI-based solution.
4. The stakeholders should also ensure that gambling is conducted in a social setting, with family, friends or colleagues to keep gamblers accountable for their limits and ensure gambling is done for limited amounts of time, both in frequency and duration.

### 6.3 Contribution to knowledge

This study contributes a novel AI-based solution that can classify gambling activities. The proposed solution provides insights that can assist operators or regulators prevent irresponsible behaviours during gambling activities.

It contributes to safe gambling and promotes the integrity of gambling operators. Furthermore, contributes to more entertaining and enjoyable gambling activities through harm minimisation strategies. The implementation of the artificial intelligence model developed in this study will mitigate the consequences of irresponsible gambling, such as unhealthy emotions, excessive time spent on gambling, exceeding gambling limits through excessive betting, depression, stress and anxiety. When the gamblers become responsible in their understanding and approach to betting, the family will be happier and healthier, and the society will be more productive and safer from social ills such as crime and suicide attempts.

Methodologically, this paper demonstrates the implementation of data balancing using the SMOTE technique to enable accurate classification for minority classes. It also conducts detailed statistical analysis of gambling activities by frequency and programme. It further demonstrates the use of the AI model for gambling activities classification. The outcome of this study can serve as a decision support that can assist operators, regulators or online gambling platforms identify high-risk players and supports targeted interventions for responsible gambling.

Additionally, this research aims to raise awareness and increase knowledge about problem gambling. The system currently in use is not adequately designed to produce the much-needed results for problem gambling.

### 6.4 Limitations of the study and direction for future studies

This study is limited to the use of secondary data and an exploratory approach in developing the AI-based solution. Future studies could consider validating the proposed AI-based solution using a primary dataset.

Deep learning models such as the Convolutional Neural Network (CNN), Recurrent Neural Network (RNN) and Long Short-Term Memory (LSTM) on dataset with spatial and sequential dependencies for time series analysis detection and mitigation of problem gamblers. Furthermore, comparative analysis of the results obtained in this study vis-à-vis other hybrid, or ensemble or can be considered as part of future studies.

## Data Availability

The original contributions presented in the study are included in the article/supplementary material, further inquiries can be directed to the corresponding author.
